# Representation of Women in Contemporary Kidney Transplant Trials

**DOI:** 10.3389/ti.2023.11206

**Published:** 2023-04-14

**Authors:** A. J. Vinson, S. B. Ahmed

**Affiliations:** ^1^ Division of Nephrology, Department of Medicine, Dalhousie University, Halifax, NS, Canada; ^2^ Nova Scotia Health, Halifax, NS, Canada; ^3^ Cumming School of Medicine, University of Calgary, Calgary, AB, Canada; ^4^ Libin Cardiovascular Institute, Calgary, AB, Canada; ^5^ O’Brien Institute of Public Health, Calgary, AB, Canada; ^6^ Alberta Kidney Disease Network, Calgary, AB, Canada

**Keywords:** disparity, trials, gender, sex, participation

## Abstract

Women are often underrepresented in clinical trials. It is unclear if this applies to trials in kidney transplant (KT) and whether the intervention or trial focus influences this. In this study, the weighted participation-to-prevalence ratio (PPR) for women enrollees in KT trials was determined for leading medical transplant or kidney journals between 2018 and 2023 using meta-regression overall and in three sensitivity analyses by: 1) Whether the intervention involved immunosuppression; 2) Area of trial focus; rejection, cardiometabolic, infection, lifestyle, surgical; 3) Whether the intervention was medical/surgical or social/behavioral. Overall, 33.7% of participants in 24 trials were women. The overall pooled PPR for the included trials was 0.80, 95% CI 0.76–0.85, with significant heterogeneity between trials (*I* ^*2*^ 56.6%, p-value < 0.001). Women had a lower PPR when the trial involved immunosuppression (PPR 0.77, 95% CI 0.72–0.82) than when it did not (PPR 0.86, 95% CI 0.80–0.94) and were less likely to participate in trials with a medical/surgical versus behavioral intervention; the lowest PPR for women was in studies examining rejection risk (PPR 0.75, 95% CI 0.70–0.81). There is better representation of women in KT trials compared to other medical disciplines, however women remain underrepresented in transplant trials examining immunosuppression and rejection.

## Graphical Abstract

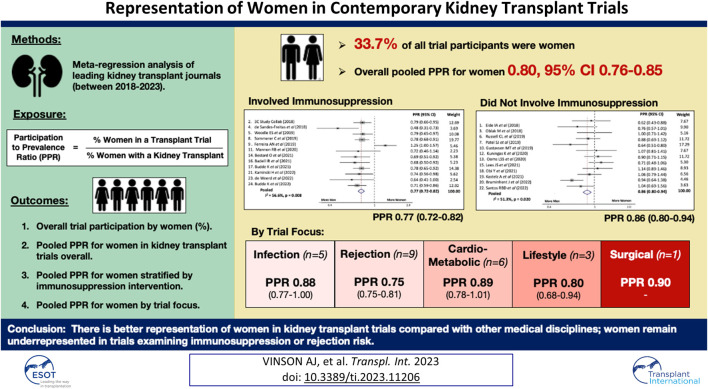

## Introduction

Sex and gender have been shown to play significant roles in kidney transplant outcomes in terms of differential immune reactivity, sensitization and rejection risk, immunosuppression medication pharmacokinetics and adherence, infectious pathogen risk, and overall graft survival (1–3). Thus, it is paramount that transplant clinical trials include appropriate representation of males and females to allow for assessment of sex and/or gender-stratified effect.

The representation of women in transplant trials can best be evaluated using the participation to prevalence ratio (PPR) which is a measure of how trial recruitment corresponds with disease or condition prevalence in the general population, i.e., the percentage of women in a trial divided by the percentage of women with a disease state in the general population, in this case, a kidney transplant. A PPR of 0.8–1.2 indicates appropriate trial representation (4, 5).

A recent study published in 2021 examined the PPR for women and minority populations in 172 abdominal transplant trials in the United States from 2000 to 2018. Compared to non-transplant studies where women have been historically and often woefully under included (4, 6–8), in abdominal transplant trials, women were surprisingly well represented (PPR 0.87) (9). Importantly however, this study did not examine trial characteristics that may have influenced female recruitment. For example, many trials in transplantation are non-interventional and whether this modified the PPR for women was not examined. Likewise, whether the area examined within transplant influences recruitment (for example, rejection, infection, cardiometabolic, adherence, etc.), remains to be seen. Previous work has suggested there may be gender differences in decision-making around trial enrollment (10). Higher proportions of female participants have been demonstrated for trials examining preventative and behavioral interventions compared with those examining treatment or medical/surgical interventions (8); whether this applies to patients with a solid organ transplant has not been previously examined.

Therefore, in this study we aimed to determine the PPR for women versus men in kidney transplant trials published in leading kidney or transplant journals over the last 5 years and determine if the PPR for women participants varied by 1) whether the intervention was related to immunosuppression or not, 2) the area of trial focus (rejection, cardiometabolic, infection, lifestyle, or surgical) and 3) whether the intervention was medical/surgical or social/behavioral.

## Methods

We included all adult kidney transplant trials published in the top 10 transplant or nephrology journals defined based on Scimago Journal and Country Rank (SJC) (11, 12) between 2018 and 2023, excluding review journals, supplements, non-kidney transplant and basic science journals. Therefore, we reviewed the American Journal of Transplantation (AJT), the Clinical Journal of the American Society of Nephrology (CJASN), Nephrology Dialysis Transplantation (NDT), Transplantation, the Clinical Kidney Journal (CKJ), Transplant International (Tx Int), the Journal of the American Society of Nephrology (JASN), Kidney International (KI), the American Journal of Kidney Disease (AJKD), the American Journal of Nephrology (AJN), and Advances in Chronic Kidney Disease (Advances in CKD). Trials were restricted to those with at least 50 participants and studies examining non-kidney transplant or simultaneous/multi-organ transplant were excluded. Within each journal’s website, articles were searched using the terms “trial” and “kidney” if it was a transplant focussed journal, or “trial” and “transplant” if it was a kidney focussed journal. We excluded any trials pertaining to waitlisted candidates not yet transplanted, donor or donor kidney interventions prior to transplant, and those looking at desensitization protocols for patients with incompatible living kidney donors given the disproportionate representation of women in this population on account of pregnancy-induced incompatibility with spouse donors (13).

While the terms sex and gender are often used interchangeably, they are not synonymous, however for this study we assumed women to mean female sex and men to mean male sex. The percentage of males and females (or men and women where indicated) in each trial was determined, and the prevalence of females in each trial was adjusted for the global prevalence of females with a kidney transplant (0.42) based on literature suggesting this is appropriate for most countries except Pakistan, India and Nepal (14).

The weighted PPR for women enrollees in kidney transplant trials was determined overall using meta-regression, and in three sensitivity analyses by:

i. Whether the intervention was related to immunosuppression or not.
ii. Area of trial focus; rejection, cardiometabolic, infection, lifestyle, surgical.
iii. Whether the intervention was medical or social/behavioral.

Heterogeneity in PPR overall and within each sensitivity subgroup was examined using the Higgins *I* ^*2*^ and chi-square test of heterogeneity (15). The proportion of trials reporting effect results in a sex-stratified analysis was also determined as was the number of trials commenting on menopausal or reproductive age status in women participants. Exclusion criteria was assessed to determine if there were barriers to enrollment specific to women of reproductive age.

## Results

We identified 25 trials conducted in kidney transplant recipients over the study period (AJT *n* = 12; Transplantation *n* = 2; KI *n* = 1; JASN *n* = 1; Tx Int *n* = 9); 1 additional AJT study was excluded on the basis of examining desensitization protocols in patients with an incompatible living donor. A flow diagram of identified trials and subsequent exclusions is shown in Supplementary Figure S1 and a summary of trial populations is presented in Supplementary Table S1.

The percentage of women in each trial ranged from 20.0% to 52.4% with 7/24 trials including less than 30% women and 18/24 trials including less than 40% women. Only 1/24 trials had ≥50% women participants. Overall, 33.7% of trial participants were women.

**FIGURE 1:**
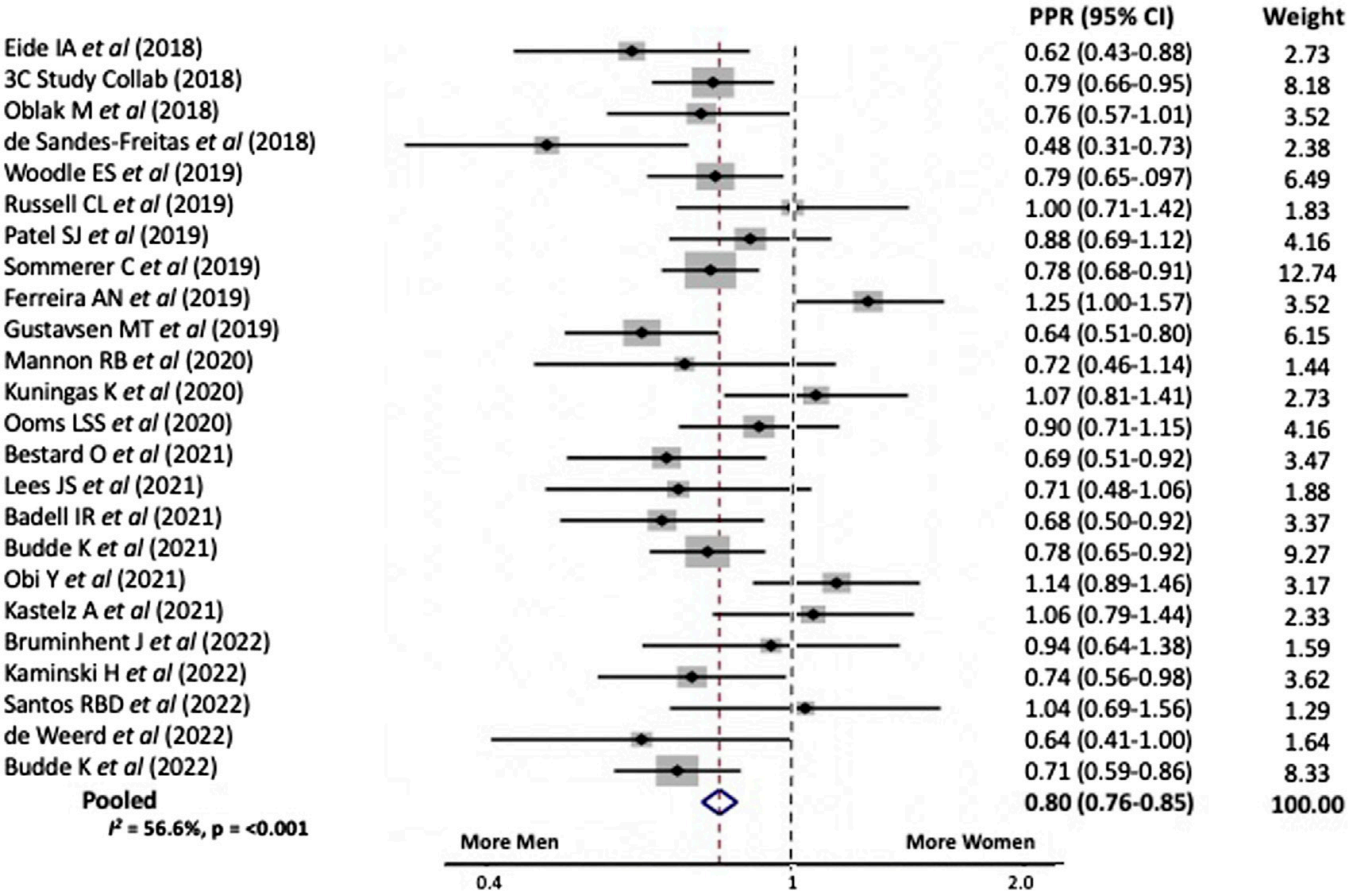
Pooled participation to prevalence ratio for women in kidney transplant trials between 2018 and 2023.

Adjusting for the global prevalence of women living with a kidney transplant, the overall pooled PPR for the included trials was 0.80, 95% confidence interval (CI) 0.76–0.85, Figure 1. There was significant heterogeneity in the PPR for the examined trials (*I* ^*2*^ 56.6%, *p*-value <0.001).

**FIGURE 2:**
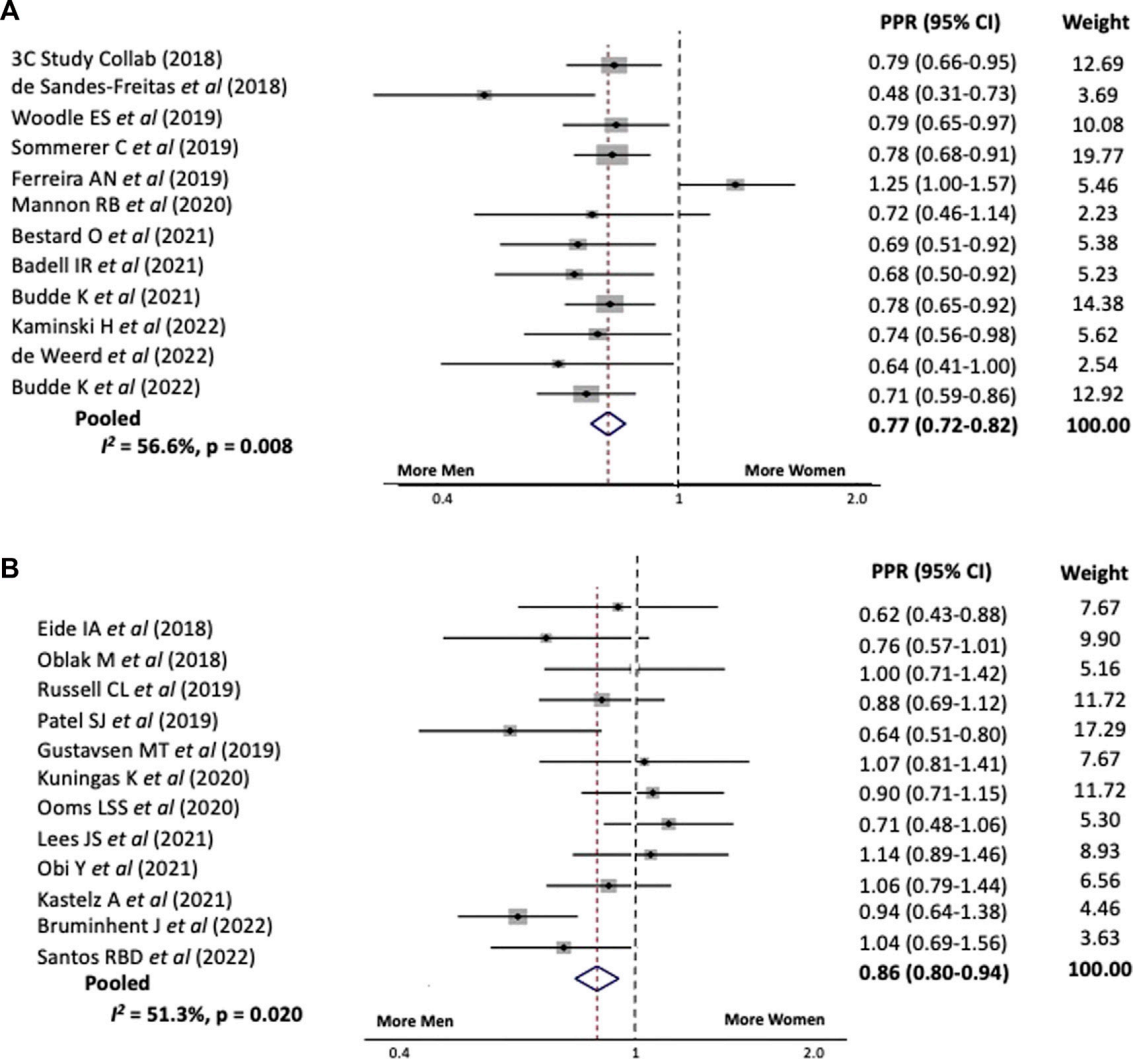
Pooled participation to prevalence ratio for women in kidney transplant trials between 2018 and 2023 stratified by whether the intervention **(A)** Involved immunosuppression or **(B)** Did not involve immunosuppression.

In sensitivity analyses we examined the PPR for the above trials stratified by the primary intervention type and study focus. When the intervention involved immunosuppression the PPR for women was 0.77, 95% CI 0.72–0.82 versus 0.86, 95% CI 0.80–0.94 when it did not, Figure 2. Study heterogeneity for both analyses was similar to that for the overall cohort.

A breakdown of trial participation by study focus demonstrated the highest PPR for women when the trial was examining surgical complications (only one study included; PPR 0.90), followed by cardiometabolic risk [PPR 0.89, 95% CI 0.78–1.01 (*n* = 6)] and infectious risk [PPR 0.88, 95% CI 0.77–1.00 (*n* = 5)], Supplementary Figure S2. The lowest PPR for women was in studies examining rejection risk [PPR 0.75, 95% CI 0.70–0.81 (*n* = 9)]. Heterogeneity for PPR was significant for all subcategories of study focus except rejection risk (*I* ^*2*^ 0.0%, *p*-value 0.953) suggesting *consistent* underrepresentation of women in these trials (26.6%–33.2% women participants). When the intervention was medical (a medication or surgical intervention) the PPR for women was 0.80, 95% CI 0.75–0.84, whereas behavioural or lifestyle intervention trials had a slightly higher PPR for women of 0.86, 95% CI 0.74–0.98, Supplementary Figure S3.

Of the 24 studies examined, 2 presented results in a sex-stratified manner and 0/24 commented on menopausal or reproductive age status for women trial participants. Seven excluded pregnant or lactating women, or women of childbearing potential unless using effective methods of contraception, and 1 study listed “breastfeeding or of childbearing potential” as an exclusion with no further explanation, Supplementary Table S1.

## Discussion

In this study, we demonstrate better representation of women in kidney transplant trials compared to what has been shown for other medical disciplines, confirming Zaldana et al’s earlier findings (9). Overall, we demonstrate a pooled PPR of 0.80 (a PPR of 0.8–1.2 indicates appropriate trial representation) (4, 5) which is significantly better than our earlier examination of the PPR for women in recent non-transplant clinical trials examining medications with important cardiorenal indications (PPR 0.70 for sodium glucose cotransporter-2 inhibitors, 0.72 for glucagon-like peptide-1 receptor agonists, and 0.56 for non-steroidal mineralocorticoid receptor antagonists) (6).

However, there were important gender differences in transplant trial participation based on the trial’s aim. When we examined the involvement of women in trials by intervention type, the PPR for women was lower when the study examined changes to immunosuppression (0.77 for immunosuppression trials versus 0.86 for other interventions). Similarly women were better represented in trials that examined the outcome of infectious risk (PPR 0.88), cardiometabolic risk (PPR 0.89), or surgical complications (PPR 0.90; only one trial) compared with a rejection outcome (PPR 0.75). Finally, trials examining a social or behavioural intervention included more women than those examining medical or surgical interventions (PPR 0.86 versus 0.80).

Potential reasons for these differences require further investigation. Female kidney transplant recipients are at higher risk for transplant rejection and death-censored graft loss (1) relating to sex-based differences in immunosuppression pharmacokinetics and pharmacodynamics (16, 17), gender-related differences in medication adherence (18), genetic and estrogen-related stimulation of the immune response (19, 20) and other less defined mechanisms. Importantly, existing common immunosuppressive therapies including mycophenolic acid (21, 22) and tacrolimus (23–25) have shown significant differences in clearance and metabolism by sex, with differential drug concentrations and side effects noted in women and men on equivalent doses. Therefore, the fact that recent clinical transplant trials examining rejection risk and immunosuppressive therapies included the lowest proportion of women (below what is considered an acceptable PPR range) is a major concern. Furthermore, given the changes in sex hormone expression (and thereby immune response) over the lifespan, recipient current age has been shown to modify the association between recipient sex and transplant outcomes (1, 19). There is a drop in sex hormone levels in post-menopausal women which associates with less immune reactivity and thereby rejection risk compared with women of reproductive age (26, 27). However, despite the influence menopause status has been shown to have on transplant rejection risk, menopause status was not mentioned in any of the 24 trials included in this meta-regression.

Why women are better represented in kidney transplant trials than in studies of other medical disciplines is unknown. Women have been shown to be more risk averse than men and demonstrate a greater perception of harm associated with trial participation, resulting in a corresponding reluctance to enrol in clinical trials (28, 29). However, kidney transplant recipients may represent a biased population of women who are more risk tolerant in so far as they accepted the potential risk of kidney transplant, and thus may similarly be more willing to participate in clinical trials. Another potential explanation is that there is a relative paucity of evidence in the kidney transplant population (30) and therefore more equipoise regarding the benefit with currently accepted standards of care. This may result in less perception of risk with trial enrollment; this requires further study. Furthermore, women make informed decisions differently from men; they spend more time gathering information before signing a consent, and they rely on different sources (medical and non-medical) and often seek advice from family members or friends (10, 31). A study of American transplant clinicians identified adequate social supports as the second most important factor to define transplant eligibility (32); therefore transplant may select for a subset of women with social supports to facilitate discussions, and potentially reassurance, regarding trial participation.

Importantly with the literature available, we are unable to ascertain whether the barrier to trial participation is that women are not being approached and consented for enrollment at the same rate as their male counterparts, or if women are being approached but declining involvement. This is a critical first step to ensuring equitable representation in trials by gender. In the included studies, 29.2% listed breastfeeding, pregnancy or childbearing potential without efficient contraception as exclusions. Importantly, 1 study listed “breastfeeding or of childbearing potential” as an exclusion criteria which may have systematically biased against the recruitment of women of reproductive age.

Given the potential for sex and/or gender differences in drug effect or complications, appropriate representation of women in clinical transplant trials is imperative, particularly since rejection risk and immunosuppression metabolism is known to vary by sex. Studies should be adequately powered to examine potential sex-by-treatment interactions and sex-stratified analyses should be reported. Only 2 of the 24 trials included presented a sex-stratified supplementary analysis. Potential strategies to improve recruitment of women in clinical trials have been previously published (6, 33, 34) and include actionable items at the government, industry, researcher, journal, and patient level. These include, but are not limited to, ensuring gender sensitive recruitment and communication tools, targeted recruitment of women and gender diverse participants, and the inclusion of more women and gender diverse researchers on study teams, patient advisory boards, and in leadership positions for regulatory agencies and pharmaceutical companies (6). However, even when studies have pre-specified aims to recruit 50% women, women are often still underrepresented in the final results as demonstrated in the Action to Control Cardiovascular Risk in Diabetes (ACCORD) trial which planned to include 50% women, but ultimately included only 38.4% women (35). Importantly, lack of adequate diversity amongst trial constituents can result in an inability to identify treatment effect in specific trial populations (including women); extrapolation of data from one population to another may not be appropriate particularly in the face of substantial biologic or sociodemographic differences.

In light of the perpetual underrepresentation of women in clinical trials, policymakers have examined strategies to bolster trial recruitment of women (36, 37). The Sex and Gender Equity in Research (SAGER) guidelines provide recommendations for the reporting of sex and gender in medical research and were developed based on a recognition of sex and/or gender differences in disease prevalence, outcomes, and response to therapy. A 2021 letter to editor published in Transplantation in 2021 highlighted the fact that while an increasing number of science and medical journals were endorsing the SAGER guidelines, no transplant focussed journals had pledged to the same (38). In response, *Transplantation* now includes a link to the SAGER guidelines in their instructions to authors, however it is as of yet too early to tell whether this has improved sex and/or gender-based reporting.

While this study contributes to the growing body of literature surrounding equitable representation of women in clinical trials, there are important limitations. First, this study identified clinical trials published in the top 10 transplant or nephrology journals defined using SJC over the study period using discrete search terms on the journal’s website; “kidney” + “trial” for transplant journals and “transplant” + “trial” for kidney journals. Thus, while we anticipate most, it not all, relevant clinical trials would be identified in this manner, it is possible there were otherwise appropriate trials that did not meet our search criteria that were not included. However, we would expect such trials to be missing at random and unlikely to significantly deviate from or impact our pooled PPR results. Secondly, the PPR in and of itself has limitations. The base population prevalence used was an average global prevalence for women living with kidney transplant based on 2016 data. There has been little change in the proportion of women versus men transplanted over time, and this value is felt to appropriately reflect the prevalence of women with a kidney transplant in most countries especially over this contemporary timespan, however there are likely small degrees of geographic variability not accounted for (14). That said, no trial was conducted in one of the three countries noted to have a disproportionately low rate of transplantation in women versus men in the above study (e.g., Pakistan, India or Nepal) (14). Finally, there are a paucity of clinical trials occurring in the kidney transplant population. This entire analysis included 4,811 participants over 24 studies. For comparison, the recently published EMPA-KIDNEY trial examining Empagliflozin in patients with chronic kidney disease randomized 6,609 patients in and of itself. Although we restricted our study to include only trials with at least 50 participants, many individual trials were relatively small hence why we performed a weighted meta-regression to create a pooled PPR for the primary and each secondary subgroup analyses; only one trial had a surgical focus (*n* = 200) and thus the results for this subgroup PPR must be interpreted with caution.

In order to generate evidence in kidney transplant patients that applies to both men and women, participants of all genders must be represented in clinical transplant trials with appropriate sex stratification in analysis and reporting of results. This requires women of all ages be approached for recruitment and not disproportionately excluded from participation, and importantly, women have to be willing to partake. Strategies to not only increase the inclusion of women in trials, but also to collect female sex-specific factors have been outlined elsewhere (39, 40). Fortunately the representation of women in kidney transplant trials appears to be better than for other fields in medicine (4, 6–8). Whether transplant researchers are intentionally more inclusive with recruitment, or women living with a kidney transplant are more willing to participate in trials remains to be seen. Importantly despite this, women remain underrepresented in kidney transplant trials examining rejection and immunosuppression therapies; both areas where patient sex modifies risk. Thus, despite advances in inclusivity in transplant studies relative to other genres of medicine, there are still gains to be made.

## Data Availability

The original contributions presented in the study are included in the article/Supplementary Material, further inquiries can be directed to the corresponding author.
