# Supplementary material for: Representation of Women in Contemporary Kidney Transplant Trials
Source: Transpl Int. 2023 Apr 14;36:11206. doi: 10.3389/ti.2023.11206 (PMC10141646; doi:10.3389/ti.2023.11206)
Supplement: Supplementary file 1 [file DataSheet1.docx]

**Supplemental Table 1: Study characteristics**

| **No** | **Title** | **Journal** | **Pub**  **Date** | **Study Focus** | **Intervention** | **% Females** | **Sex-Stratified Analysis** | **Exclusion Criteria** |
| --- | --- | --- | --- | --- | --- | --- | --- | --- |
| **1** | ***Eide IA, et al***  Effects of marine n-3 fatty acid supplementation in renal transplantation: A randomized controlled trial | AJT | 2018 | Non-immunosuppression graft function | 2.6 g of marine n‐3 fatty acids versus olive oil | 25.8% | N | N/A |
| **2** | ***The 3C Study Collaborative Group***  Campath, calcineurin inhibitor reduction, and chronic allograft nephropathy (the 3C Study) – results of a randomized controlled clinical trial | AJT | 2018 | Immunosuppression | Alemtuzumab versus basiliximab induction therapy; tacrolimus versus sirolimus maintenance therapy | 33.0% | Y | N/A |
| **3** | ***Oblak M et al***  Paricalcitol versus placebo for reduction of proteinuria in kidney transplant recipients: a double-blind, randomized controlled trial | Tx Int | 2018 | Proteinuria | 24 weeks’ treatment with 2 g/day paricalcitol versus placebo | 32.1% | N | Pregnancy; Breastfeeding |
| **4** | ***de Sandes-Freitas et al***  The impact of everolimus in reducing cytomegalovirus events in kidney transplant recipients on steroid-avoidance strategy: 3-year follow-up of a randomized clinical trial | Tx Int | 2018 | Cytomegalovirus | Everolimus versus mycophenolate sodium | 20.0% | N | N/A |
| **5** | ***Woodle ES et al***  Belatacept-based immunosuppression with simultaneous calcineurin inhibitor avoidance and early corticosteroid withdrawal: A prospective, randomized multicenter trial | AJT | 2019 | Immunosuppression | Alemtuzumab/belatacept versus ATG/belatacept versus ATG/tacrolimus | 33.2% | N | Pregnancy; no contraception |
| **6** | ***Russell CL et al***  Improving medication adherence and outcomes in adult kidney transplant patients using a personal systems approach: SystemCHANGE™ results of the MAGIC randomized clinical trial | AJT | 2019 | Adherence | System CHANGE intervention versus attention control (patient education) * | 41.6% | N | N/A |
| **7** | ***Patel SJ et al***  Ciprofloxacin for BK viremia prophylaxis in kidney transplant recipients: Results of a prospective, double-blind, randomized, placebo-controlled trial | AJT | 2019 | BK viremia | 3‐month course of ciprofloxacin versus placebo | 37.0% | N | N/A |
| **8** | ***Sommerer C et al***  An open-label, randomized trial indicates that everolimus with tacrolimus or cyclosporine is comparable to standard immunosuppression in de novo kidney transplant patients | KI | 2019 | Immunosuppression | Everolimus + Tacrolimus versus Everolimus + Cyclosporine versus Mycophenolic acid + Tacrolimus | 32.8% | N | Pregnancy; Breastfeeding; no contraception |
| **9** | ***Ferreira AN et al***  Prospective randomized study comparing everolimus and mycophenolate sodium in de novo kidney transplant recipients from expanded criteria deceased donor | Tx Int | 2019 | Cytomegalovirus | ATG + delayed introduction of reduced dose tacrolimus, prednisone and everolimus versus mycophenolate | 52.4% | N | N/A |
| **10** | ***Gustavsen MT et al***  Evaluation of tools for annual capture of adherence to immunosuppressive medications after renal transplantation – a single-centre open prospective trial | Tx Int | 2019 | Adherence | Intensive or single-point adherence assessment * | 26.8% | N | N/A |
| **11** | ***Mannon RB et al***  Avoidance of CNI and steroids using belatacept—Results of the Clinical Trials in Organ Transplantation 16 trial | AJT | 2020 | Immunosuppression | Belatacept (ATG) versus Tacrolimus (ATG) versus Belatacept + Tacrolimus (Basilix) | 30.4% | N | Unexplained stillborn or spontaneous abortion |
| **12** | ***Kuningas K et al***  Comparing Glycaemic Benefits of Active Versus Passive Lifestyle Intervention in Kidney Allograft Recipients: A Randomized Controlled Trial | Tx | 2020 | Insulin secretion | Lifestyle advice delivered by renal dietitians using behavior change techniques versus leaflet advice alone * | 45.4% | N | N/A |
| **13** | ***Ooms LSS et al***  Stenting the ureteroneocystostomy reduces urological complications in kidney transplantation: a noninferiority randomized controlled trial, SPLINT trial | Tx Int | 2020 | Surgical complication | Ureteric stent versus no stent | 38.0% | N | N/A |
| **14** | ***Bestard O et al***  Preformed T cell alloimmunity and HLA eplet mismatch to guide immunosuppression minimization with tacrolimus monotherapy in kidney transplantation: Results of the CELLIMIN trial | AJT | 2021 | Immunosuppression | Tacrolimus monotherapy versus Tacrolimus-based standard of care | 28.7% | N | Pregnancy; Breastfeeding; no contraception |
| **15** | ***Lees JS et al***  The ViKTORIES trial: A randomized, double-blind, placebo-controlled trial of vitamin K supplementation to improve vascular health in kidney transplant recipients | AJT | 2021 | Vascular Health | Vitamin K supplementation versus placebo | 30.0% | N | Breastfeeding or of childbearing potential |
| **16** | ***Badell IR et al***  Every 2-month belatacept maintenance therapy in kidney transplant recipients greater than 1-year posttransplant: A randomized, noninferiority trial | AJT | 2021 | Immunosuppression | Belatacept q1m versus q2m | 28.2% | N | N/A |
| **17** | ***Budde K et al***  Conversion from Calcineurin Inhibitor– to Belatacept-Based Maintenance Immunosuppression in Renal Transplant Recipients: A Randomized Phase 3b Trial | JASN | 2021 | Immunosuppression | Switch to Belatacept versus continue calcineurin inhibitor | 32.5% | Y | N/A |
| **18** | ***Obi Y et al***  Correcting anemia and native vitamin D supplementation in kidney transplant recipients: a multicenter, 2 3 2 factorial, open-label, randomized clinical trial | Tx Int | 2021 | Non-immunosuppression graft function | High versus low hemoglobin target and cholecalciferol 1000 IU/day versus placebo | 47.7% | N | Pregnancy; Breastfeeding |
| **19** | ***Kastelz A et al***  Personalized physical rehabilitation program and employment in kidney transplant recipients: a randomized trial | Tx Int | 2021 | Exercise; employment | 12-month exercise rehabilitation program versus standard care alone * | 44.2% | N | N/A |
| **20** | ***Bruminhent J et al***  An additional dose of viral vector COVID-19 vaccine and mRNA COVID-19 vaccine in kidney transplant recipients: A randomized controlled trial (CVIM 4 study) | AJT | 2022 | COVID vaccine | Pfizer versus Moderna mRNA vaccine | 39.0% | N | N/A |
| **21** | ***Kaminski H et al***  Incidence of cytomegalovirus infection in seropositive kidney transplant recipients treated with everolimus: A randomized, open-label, multicenter phase 4 trial | AJT | 2022 | Cytomegalovirus | Everolimus versus mycophenolic acid | 31.0% | N | Pregnancy; no contraception |
| **22** | ***Santos RBD et al***  Sitagliptin Versus Placebo to Reduce the Incidence and Severity of Posttransplant Diabetes Mellitus After Kidney Transplantation—A Single-center, Randomized, Double-blind Controlled Trial | Tx | 2022 | Post-transplant diabetes | Sitagliptin versus placebo | 44.3% | N | N/A |
| **23** | ***de Weerd AE et al***  Tacrolimus Monotherapy is Safe in Immunologically Low-Risk Kidney Transplant Recipients: A Randomized-Controlled Pilot Study | Tx Int | 2022 | Immunosuppression | Tacrolimus/mycophenolate mofetil continued versus taper and discontinue MMF at month 9 | 26.6% | N | No contraception |
| **24** | ***Budde K et al***  Prolonged-Release Once-Daily Formulation of Tacrolimus Versus Standard-of-Care Tacrolimus in *de novo* Kidney Transplant Patients Across Europe | Tx Int | 2022 | Immunosuppression | Immediate-release tacrolimus versus prolonged-release tacrolimus | 29.7% | N | N/A |

*American Journal of Transplantation (AJT), Tx (Transplantation), KI (Kidney International), Journal of the American Society of Nephrology (JASN), Transplant International (Tx Int), ATG (anti-thymocyte globulin), MMF (mycophenolate mofetil)*

**Behavioral/lifestyle Intervention*

**Supplemental Figure 1:** Flow Diagram of the Transplant and Nephrology Journals Included in the Meta-Regression. *American Journal of Transplantation (AJT); Kidney International (KI); Journal of the American Society of Nephrology (JASN); Clinical Journal of the American Society of Nephrology (CJASN); Transplant International (Tx Int); Clinical Kidney Journal (CKJ); Nephrology Dialysis Transplantation (NDT); American Journal of Kidney Disease (AJKD); American Journal of Nephrology (AJN), Advances in Chronic Kidney Disease (Advances in CKD)*

**
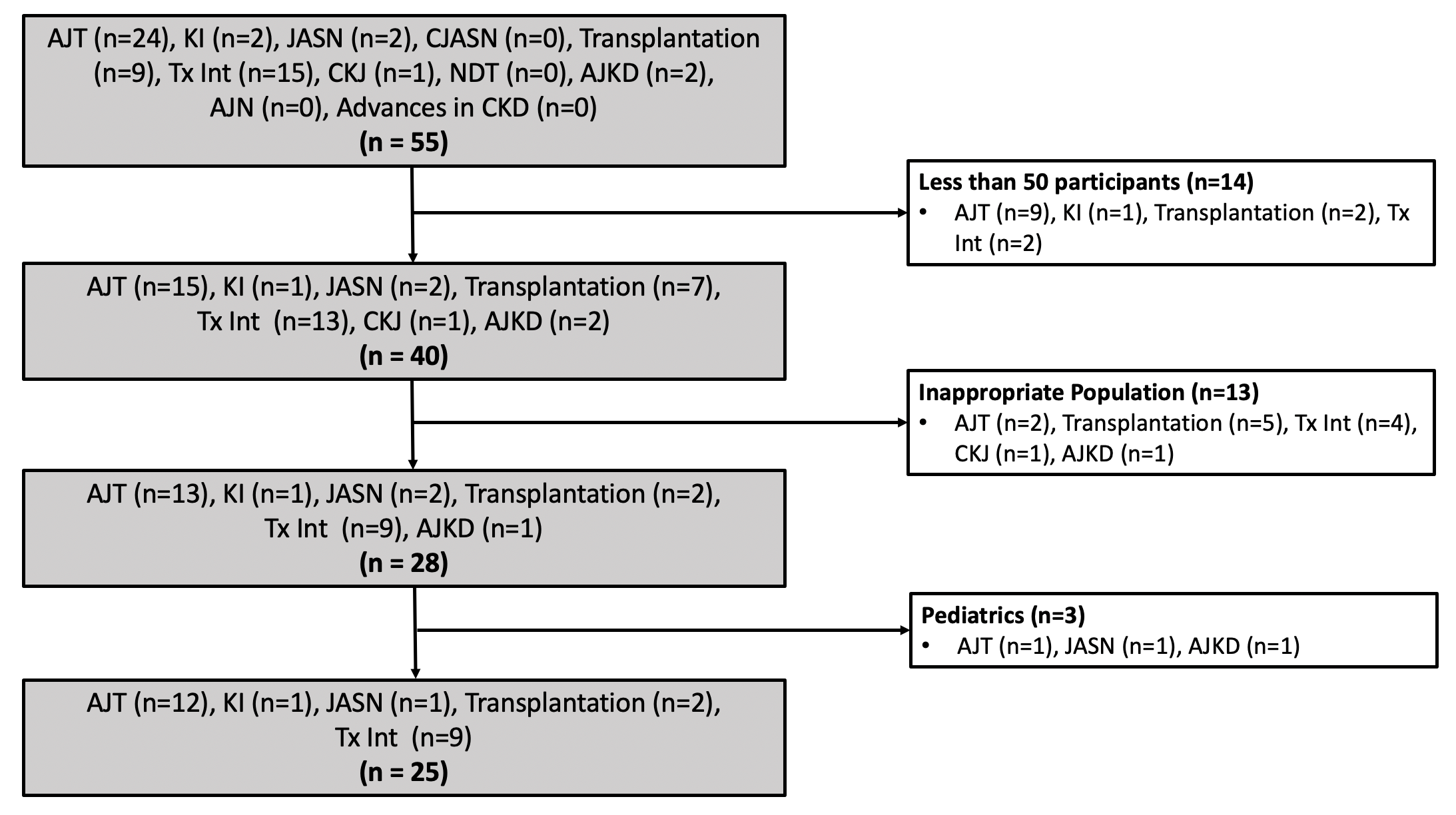
**

**Supplemental Figure 2:** Pooled Participation to Prevalence Ratio for Women in Kidney Transplant Trials Between 2018 and 2023 Stratified by Trial Focus: a) infection; b) rejection; c) cardiometabolic; d) lifestyle; e) surgical

**a) Infection**

**
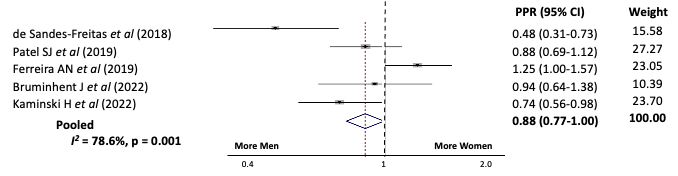
**

**b) Rejection**

**
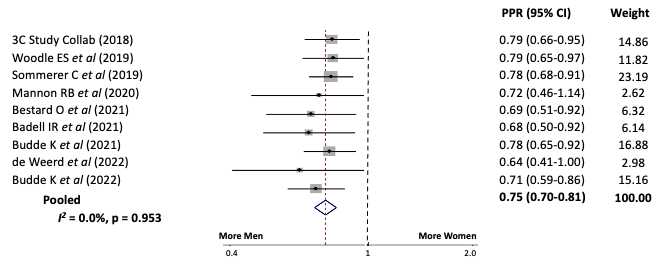
**

**c) Cardiometabolic**

**
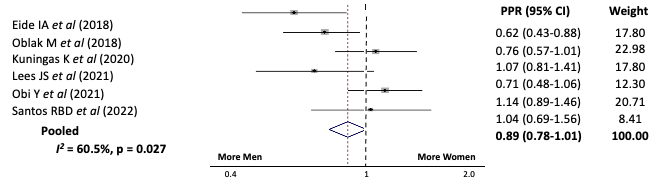
**

**d) Lifestyle**

**
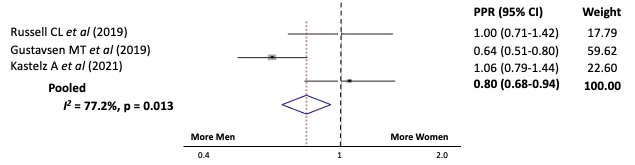
**

**e) Surgical**

**
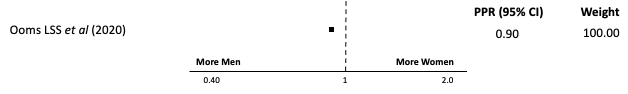
**

**Supplemental Figure 3:** Pooled Participation to Prevalence Ratio for Women in Kidney Transplant Trials Between 2018 and 2023 Stratified by Type of Intervention: a) medical or surgical; b) behavioral/lifestyle

**a) Medical or Surgical Intervention**

**
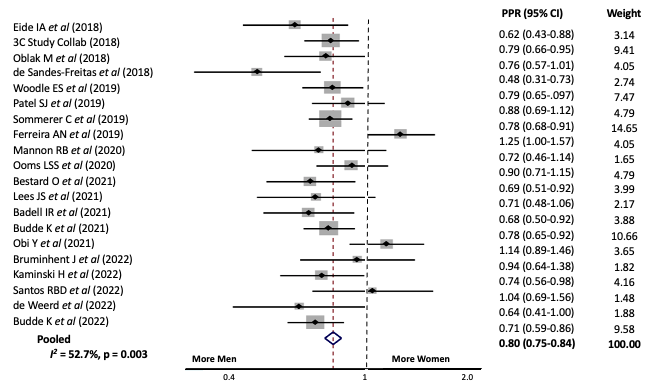
**

**b) Behavioral or Lifestyle Intervention**

**
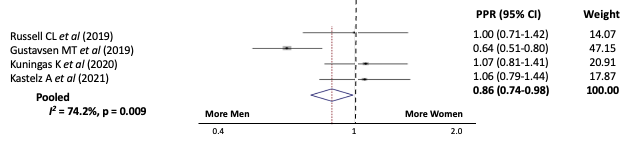
**
